# Genetic Testing in Familial Hypercholesterolemia: Is It for Everyone?

**DOI:** 10.1007/s11883-023-01091-5

**Published:** 2023-03-02

**Authors:** A. M. Medeiros, M. Bourbon

**Affiliations:** 1 Unidade de I&D, Grupo de Investigação Cardiovascular, Departamento de Promoção da Saúde E Prevenção de Doenças Não Transmissíveis, Instituto Nacional de Saúde Doutor Ricardo Jorge, Lisbon, Portugal; 2 BioISI – Biosystems & Integrative Sciences Institute, Faculdade de Ciências, Universidade de Lisboa, Lisbon, Portugal

**Keywords:** Familial hypercholesterolemia, FH, Dyslipidemia, LDLR, FH screening, High cholesterol

## Abstract

**Purpose of Review:**

Lipid measurements and genetic testing are the main diagnostic tools for FH screening that are available in many countries. A lipid profile is widely accessible, and genetic testing, although available worldwide, in some countries is only performed in a research context. Still FH is diagnosed late, showing lack of early screening programs worldwide.

**Recent Findings:**

Pediatric screening of FH was recently recognized by the European Commission Public Health Best Practice Portal as one on the best practices in non-communicable disease prevention. The early diagnosis of FH and the lowering of LDL-C values over lifespan can reduce the risk of coronary artery disease and offer health and socioeconomic gains.

**Summary:**

Current knowledge about FH shows that early detection through appropriate screening needs to become a priority in healthcare systems worldwide. Governmental programs for FH identification should be implemented to unify the diagnosis and increase patient identification.

## Introduction

Familial hypercholesterolemia is the most common monogenic disorder (1:300) [1], characterized by lifelong exposure to elevated low-density lipoprotein cholesterol (LDL-C) levels that accelerate the atherosclerotic process and consequently increases the risk of coronary artery disease (CAD) [2, 3]. Although frequent, FH is underdiagnosed and undertreated in most countries [4]. The diagnosis is made late, on average after the age of 40 years and only 2% before 18 years, showing lack of early screening programs worldwide [5••].

Lipid measurements and genetic testing are the main diagnostic tools for FH screening. The determination of the lipid profile is widely accessible, and while often incorporated into routine health maintenance visits in adults, it is infrequently performed in children. Genetic testing is not widely implemented, mainly due to its cost; however, in a research context, genetic testing is performed worldwide [4]. The treatment for FH has been available for many years and reported to be safe and well-tolerated in children and adults as demonstrated in follow-up studies [6•, 7, 8]. The early diagnosis of FH and the lowering of LDL-C values over the lifespan reduces CAD risk and offers health and socioeconomic benefits [9, 10, 11••, 12]. The genetic diagnosis can add important information for the stratification of the patient’s cardiovascular risk and treatment optimization [13••]. For all these reasons, FH screening is considered as a good example where personalized medicine can be achieved [14] (https://www.icpermed.eu/en/best_practice_examples.php), and pediatric screening of FH was recently recognized by the European Commission Public Health Best Practice Portal as one on the best practices in non-communicable disease prevention [15•, 16].

## FH Screening

Screening is necessary to improve the low diagnostic rates of FH across the different countries. Different strategies can be implemented, and the best FH screening strategy depends on the healthcare system characteristics of each country [17••]. As reported in the Best Practice Portal, different types of screening may be conducted: cascade screening or reverse cascade screening, selective or opportunistic screening, and universal screening. In Europe, pediatric FH diagnosis occurs mostly by testing children of FH parents, and there are few population-based screening programs for FH currently underway [11••, 15•]. One exception is Portugal where about 40% of the FH cohort are index children with a clinical diagnosis of FH [11••, 18, 19].

The first two screenings strategies successfully implemented, in Europe, to detect FH in children and adolescents were the cascade screening in the Netherlands in 1994 [20] and the universal pediatric screening for FH in Slovenia in 1995 [21]. Other countries are starting pilot tests to implement, in a further stage, a national FH pediatric screening, as Austria [22], Germany [23], and Slovakia [15•].

1. *Cascade Screening Model*

The cascade screening model in the Netherlands was a governmental-funded program and relied upon identification of index patients (including genetically confirmation) followed by cascade screening of their first-degree relatives, from the age of 6 onwards, and then second and third-degree relatives. Over 20 years, the program has led to the identification of over 30,000 genetically confirmed FH cases (53% of the expected number of FH patients), but after the government-funded program ended in 2014, the number of new FH cases identified decreased substantially [20]. Although in a smaller scale, other similar cascade screening programs have been successfully implemented in several other European countries like Norway [24], Czech Republic [25], Spain [26], and the UK [27]. In the Czech Republic, a program, based on the MedPed (Make Early Diagnoses to Prevent Early Deaths) project, combining selective and cascade screening was implemented in 1998, and by 2017, they have identified 17.4% of the estimated number of existing FH cases in the country, based upon an FH prevalence of 1:250 [25].

2. *Selective Screening and Other Initiatives*

Both the American Academy of Pediatrics (AAP) and the Expert Panel of the National Heart, Lung, and Blood Institute (NHLBI) recommend selective pediatric lipid screening to detect FH, in children with a family history of early atherosclerotic disease or high cholesterol [28, 29].

In Austria, a selective screening method was implemented in primary schools (5 to 7 years old) to identify children with FH [22]. A total of 18,152 children were examined between January and May 2017 using a questionnaire to identify a “positive” family history. From the 229 positive questionnaires, only 133 children agreed to undergo a blood test as well as their siblings. This selective screening method identified nine children with elevated LDL-C and an additional four siblings from the 85 tested. The diagnosis was genetically confirmed in 4 index and in one sibling.

However, selective screenings can miss 30 to 60% of children with elevated LDL-C because they have no family history of early heart disease [30] or young parents may never have undergone cholesterol testing in their lives.

Because family histories may sometimes be inaccurate, incomplete or absent, the Expert Panel of the National Heart, Lung, and Blood Institute (NHLBI) strongly recommends cholesterol screening by primary care providers for all children, once between the ages of 9 and 11 years and again between 18 and 21 years [29, 30].

Based on these recommendations, the Portuguese Directorate General of Health (DGS) strongly recommends not only a selective lipid screening in children (between 2 and 4 years old) with a family history of premature CAD or high cholesterol, but also opportunistic lipid screenings, twice in pediatric age, preferably the first before the age of 10 [31].

3. *Universal Pediatric FH Screening Model*

Universal lipid screening identifies children with either a modest or more marked elevations in LDL-C, who do not meet the criteria for selective testing screening [32].

The first universal pediatric FH screening model was established in 1995 in Slovenia. The program consisted of a two-step approach: universal hypercholesterolemia screening (1st step) in pre-school children (5 years old) at their programmed visit at the primary care pediatrician, where total cholesterol measuring was mandatory, followed by genetic FH testing (2nd step) in children with elevated total cholesterol (> 5.5 mmol/L). These children were then referred to the tertiary care level (lipid clinic) [21]. More recently, the program was updated to a universal 3-step FH pediatric screening approach and includes a reverse cascade testing (study of the parent with high LDL-C level or both parents if unclear, and siblings). This universal FH screening reached > 91% of the pediatric population and enabled the detection of children and their parents’ screening at reasonable costs. In fact, in > 90% of the families, the parent with reported higher cholesterol levels was positive for the familial genetic variant [33]. Another example of child-parent FH screening in primary care was reported in 2016 by Wald and collaborators [34]. In this program, capillary blood samples were used to measure cholesterol levels in children aged 1–2 years during routine immunization visits in primary care. Children with total cholesterol values above the 95th percentile undergone genetic testing. With this approach, for every 1000 children screened, they identified 4 FH children and 4 parents with FH. The greater relevance of this child-parent screening at this age (1–2 years old) is that it avoids a separate clinic visit and offers screening at a time when parents are particularly receptive to preventing disease in their child [34]. In Australia, a pilot study conducted between 2018 and 2020 using the same approach as Wald’s program revealed it to be a potentially cost-effective detection strategy for families at risk of FH. In this program, 448 children were screened and 32 (7.1%) were found to have total cholesterol above 5.3 mmol/L. The FH diagnosis was then confirmed in 3 children, and reverse cascade testing enabled the identification of 5 additional individuals with FH [35]. More recently, Czech Republic initiated a pilot program for universal screening of lipids from cord blood followed by genetic testing for those with cholesterol above the 85th percentile [15•].

## Recommendations for FH Screening

The cascade screening program alone cannot identify all patients in each country since it requires a separate method to identify new FH index cases. A combination of pediatric universal cholesterol screening followed by genetic testing and reverse cascade testing for FH has been shown to be cost-effective in the UK [36]. The advantages of universal screening in children are the ability to perform reverse cascade screening of the parents and to initiate preventive treatment earlier, including implementation of healthy lifestyles, at the age that will provide the most benefit for the future health of the child. In adulthood, FH identification in some cases is much less effective because 20% of index cases already had premature cardiac events and the identification of FH is more difficult since there are many other reasons for high cholesterol [5••, 17••, 19, 37].

In October 2021, a first technical meeting on pediatric FH screening in Europe was organized by FH Europe under the Slovenian EU Presidency, and in September 2022, a second meeting was held under the Czech EU Presidency. These meetings brought together policymakers and the FH community with the aim of elaborating a professional consensus on pediatric FH screening in Europe (https://www.fhscreening2022.eu/) [15•]. Following this meeting, several countries endorsed the Prague Declaration, a call to action for the implementation of FH pediatric screening in Europe, addressed to national and European Union policymakers and decision-makers [38••]. The Prague Declaration proposes a set of policy recommendations to address FH screening as follows:

1. National political leaders should commit to make FH pediatric screening a reality in their country.
2. National governments should mobilize the required investment and create a policy framework for raising awareness of FH among medical practitioners and the public and build trust and responsiveness.
3. Every country should establish systematic early detection screening and diagnosis for FH, with an appropriate care program focused on children identification and treatment in accordance with the organizational structure and practices of its healthcare systems.
4. Specific actions are needed to address the barriers to successful large-scale uptake of screening programs and subsequent treatment.
5. Research should be performed to address knowledge gaps (new methods for early identification, diagnosis, personalized treatments, and follow-up; registries that document FH care, monitor progress and measure health outcomes; long-term clinical trial and longitudinal studies in children and young people; implementation studies to facilitate guideline-based FH care; personalized medicine for prevention and treatment in FH).
6. Build the capacity of healthcare professionals and empower patients on how to best support individuals and families with FH.
7. Commitment to shared learning and monitoring through international exchange and comparisons both inside and outside the EU.

Although treatment for FH children is provided by specific guidelines and recommended to be initiated at 8–10 years of age [9, 11••], the optimal age for a universal screening for FH is yet to be determined. NICE guidelines suggest that children aged 0–10 years at risk of inherited FH from one affected parent should be tested at the earliest opportunity [39]. But since the basis of heterozygous FH management is a healthy lifestyle from a toddler age and statin treatment from 8 to 10 years, Wiegman [9] recommended to screen children with suspected FH from 5 years of age or earlier if homozygous FH is suspected (both parents affected or xanthoma present). The universal FH screening in Slovenia approach also chose the age of 5 since atherosclerotic process may start in childhood and could be significantly modified by early intervention [21].

The FH Best Practice from the European Commission Public Health Portal advocates that the optimum target population is pre-pubertal (children) because they are highly motivated and can easily incorporate changes toward a healthy lifestyle (diet and exercise) and the second best option is pubertal (adolescents) already adversely affected by lipid disorders [16].

## Reasons for Genetic Testing

A diagnosis of FH can be made based on clinical findings alone using different clinical criteria (Dutch Lipid Clinic Network Diagnostic Criteria or the Simon Broome Register Diagnostic Criteria or MEDPED); however, the genetic testing of FH provides a definite molecular diagnosis of FH, prognostic information, and the ability to perform refined CAD risk stratification in these patients [10]. The NICE guidelines and the European Atherosclerosis Society/European Cardiology Society dyslipidemia guidelines also recognize the clinical importance of confirming the diagnosis of FH by a genetic test and performing cascade screening using DNA testing in relatives of patients with genetically confirmed FH [39, 40]. FH genetic testing has also been shown to have a positive effect on the initiation of lipid lowering treatment, adherence to therapy, and consequently in LDL-C reduction [10]. Other evidence, reported from a patients’ perspective, recognize that the value of genetic confirmation in FH screening programs includes knowing the cause of the high cholesterol, knowing that it was not related to behavior, and that effective treatment is available and can be prescribed from early ages to prevent CAD events [15•].

Over the years, new advances in genetic testing have made FH easily to detect, and the costs have been reduced [41–43]. The use of expanded FH next-generation sequencing (NGS) panels enabled not only the identification of pathogenic variants in FH genes, *LDLR*, *APOB,* and *PCSK9,* but also in other relevant genes, such as *APOE*, *LIPA*, and *ABCG5/8* (FH phenocopy genes). By doing so, patients with other heritable lipid disorders can also be identified and receive adequate treatment to their condition, in the majority of the cases different from FH treatment [10, 44, 45]. The type of variants and functional analysis of less known variants can also provide evidence on disease severity, since it has been shown that patients with variants producing null alleles will, in the adulthood ages, lead to higher rates of CAD events [10, 13••, 46].

## Conclusions

Based on the available extensive body of research and clinical data, early screening for FH is highly recommended on a universal level as early in age as possible (in some countries taking place already at the age of 5 – Slovenia). FH is the most common inherited condition in the world with a high prevalence (1/300). Undetected and untreated, FH causes a high rate of CAD events which can be easily and effectively prevented in affected individuals and their relatives. Measurement of cholesterol levels is an easy, universally available, and inexpensive test that can identify individuals with clinical FH which can then be confirmed by genetic testing, the gold standard for FH identification. NGS has decreased the cost of genetic testing and almost all countries (except North Africa) has access to genetic testing as seen in the FH Study Collaboration effort [4]. What is lacking is the awareness and the understanding of this public health problem among policymakers as well as the political will to address and resolve in a systematic way the issue of early FH screening and detection. Several studies have proven the cost effectiveness of early FH screening, timely diagnosis, and treatment to decrease cardiovascular disease and to promote cardiovascular health among individuals with FH. The solution is governmentally supported and financed programs for FH identification to unify the diagnosis, integrated in the national health systems. At the present time, there are high standard identification tests, as well as several lipid lowering treatments available, most at a very low cost (statins) so FH screening must become a priority in health systems promoting public cardiovascular health. As seen in the FH Study Collaboration, FH patients are being diagnosed very late, after the age of 40 years, and 20% of these patients already have suffered a premature CAD event. In the past 3 years advocacy groups like FH Europe, the European network of FH patient organizations, together with international scientific experts, have made huge efforts to bring to the attention of the policymakers the need and the benefits of implementing pediatric FH screening, organizing high technical and political meetings at the European level. The outcomes of these meetings are high level public policy papers and a declaration (Prague Declaration) making FH screening a reality so that in 2–3 decades, the goal of eliminating premature CAD in individuals with FH can be reached.
